# Overexpression of suppressive microRNAs, miR-30a and miR-200c are associated with improved survival of breast cancer patients

**DOI:** 10.1038/s41598-017-16112-y

**Published:** 2017-11-21

**Authors:** Tsutomu Kawaguchi, Li Yan, Qianya Qi, Xuan Peng, Emmanuel M. Gabriel, Jessica Young, Song Liu, Kazuaki Takabe

**Affiliations:** 1 Breast Surgery, Department of Surgical Oncology, Roswell Park Cancer Institute, Buffalo, NY 14263 USA; 2 Department of Biostatistics & Bioinformatics, Roswell Park Cancer Institute, Buffalo, NY 14263 USA; 3 Department of Surgery, University at Buffalo Jacobs School of Medicine and Biomedical Sciences, The State University of New York, Buffalo, NY USA

## Abstract

Some microRNAs (miRNAs) are known to suppress breast cancer. However, whether the expressions of these tumor suppressive miRNAs translate to patient survival were not investigated in large cohort. Nine miRNAs (miR-30a, miR-30c, miR-31, miR-126, miR-140, miR-146b, miR-200c, miR-206, and miR-335) known to be tumor suppressive miRNAs in breast cancer were investigated in Genomic Data Common data portal miRNA-Seq dataset and The Cancer Genome Atlas (TCGA) (n = 1052). Of the 9 miRNAs, miR-30a, miR-30c, miR-126, miR-140, miR-206, and miR-335 were found to have significantly lower expression in breast cancer tissues compared to paired normal breast tissue. High expression of miR-30a or miR-200c was associated with significantly better overall survival (OS). Gene Set Enrichment Analysis (GSEA) demonstrated that low expression levels of miR-30a had the tendency to associate with gene enrichment of EMT, while miR-200c did not, in TCGA cohort, and our findings support the need of validation using large cohort to use miRNA as prognostic biomarker for patients with breast cancer.

## Introduction

The “Central Dogma” of cell biology suggests that genetic information flows in a unidirectional manner from DNA to messenger RNA (mRNA) and then to protein, which determines the cell function and behavior. In the human genome, more than 90% of the DNA sequence can be transcribed to RNAs, but only about 2% of RNA encodes proteins. The remaining 98%, which does not, are termed non-coding RNAs (ncRNA). In the last decade, ncRNAs have garnered increased appreciation for their important roles in regulating various biological processes^1–6^. Small ncRNA, termed microRNA (miRNA), was first reported in 1993 regarding its role in regulating lin-4 mRNA translation in *Caenorhabditis elegans* ^7,8^.

Since then, miRNA research has expanded rapidly, and many important functions of miRNA in intra- or extra-cellular regulation have been discovered. It is now known that miRNAs have two major post-transcriptional epigenomic regulatory functions: 1) translational repression of mRNA, and 2) mRNA cleavage^9^. In 2002, the first role of miRNAs in cancer was discovered. Expressions of miR-15 and miR-16 were suppressed in patient samples and cell lines of chronic lymphocytic leukemia, leading to the discovery of tumor suppressive miRNAs^10^. In 2005, He *et al*. showed that a cluster of miRNAs, the miR-17–92 polycistron, can promote tumor formation as a potential human oncogene and coined the term oncogenic miRNA^11^.

Regarding breast cancer, Iorio *et al*. first reported cancer-related miRNAs with specific breast cancer features, such as estrogen and progesterone receptor expression, tumor stage, vascular invasion, and proliferation index^12^. The roles of miRNAs in breast cancer biology have been continuously investigated^13–15^. It has been demonstrated that oncogenic miRNAs inhibit tumor suppressive genes or activate some oncogenic pathways, whereas suppressive miRNAs inhibit oncogenic gene function through post-translational modification^9,16,17^. The target gene is not a one-to-one correspondence. In other words, one miRNA targets several genes or pathways through post-translational mechanisms. It has been clearly demonstrated both *in vivo* and *in vitro* that the low levels of tumor suppressive miRNA are associated with cancer aggressiveness, such as cancer proliferation or tumor metastasis, and high levels of tumor suppressive miRNA inhibit cancer growth^17^. However, many of these reports have not been validated in large cohorts, which limit the statistical power of these studies. For instance, Tavazoie *et al*. demonstrated that miR-126 and miR-335 showed tumor suppressive function, and high expression levels of the each of these miRNAs was associated with better survival. However, the number of patients in this analysis was only 20. Recently, miR-200c was also demonstrated to have tumor suppressive function through epithelial-mesenchymal transition (EMT) in breast cancer. Song *et al*. and Damiano *et al*. reported that breast cancer patients with high expression levels of miR-200c had better prognosis. However, both of these studies also had relatively small cohorts from single institutions (n = 134 and 51, respectively).

The Cancer Genome Atlas (TCGA) is a joint collaboration of the National Cancer Institute (NCI) and the National Human Genome Research Institute of the National Institute of Health that has collected treatment naïve primary cancer samples from over 10,000 patients on over 30 tumor types and provides genomic and epigenomic data obtained by high-throughput sequencing techniques^18^. Approximately 1,100 breast cancer samples were collected in TCGA cohort^18^, which enables investigators to utilize large sample sizes and thereby have sufficient statistical power for analyses. For example, our group has recently demonstrated that angiopoietin pathway associated with poor prognosis utilizing TCGA as a representative database providing mRNA expression data and survival outcomes in breast cancer^19^.

Although there have been several reports that demonstrated that some miRNAs have tumor suppressive functions in breast cancer, their general clinical relevance remains unclear because their effects have only been studied in small cohorts. Therefore, the aim of this study was to investigate the clinical significance of tumor suppressive miRNAs in breast cancer utilizing TCGA, which is a “big data” set that provides sufficient statistical power with proven high quality genetic samples.

## Results

### Literature search to identify tumor suppressive miRNAs in breast cancer

We conducted a literature search using PubMed Central to identify tumor suppressive miRNAs in breast cancer. We identified several tumor suppressive miRNAs that have been reported by multiple groups but that lack validation using a sufficiently large cohort. We selected 9 miRNAs for our analysis: miR-30a, miR-30c, miR-31, miR-126, miR-140, miR-146b, miR-200c, miR-206, and miR-335, which have been reported to be tumor suppressive miRNAs in breast cancer (Table 1)^20–60^.

**Table 1:** Candidates of tumor suppressive miRNAs based on literature search in breast cancer.

miRNA	Target or related gene/pathway	Significant function	Reference
**miR-30a**	VIM	Inhibits cell migration and invasion	20–25
	Eya2	Suppresse cell proliferation and migration	
	MTDH	Suppresse tumor growth and metastasis	
	Slug	Suppress epithelial mesenchymal transition (EMT)	
	ITGB3	Suppress cell invasion	
	UBE3C	Suppress cell proliferation and migration	
**miR-30c**	VIM, TWF1	Suppress cell invasion	25–27
	NF-kB, TRADD, CCNE1	Negatively regulate cell cycle	
**miR-31**	RhoA	Inhibits several steps of the invasion-metastasis cascade	28–32
	WAVE3, RhoA	Reduces cancer progression and metastasis	
	GNA13	Reduce cell invasion	
	PRKCE	Sensitizes cells to apoptosis	
**miR-126**	IRS1	Suppress cancer progression	33–36
	IGFBP2, MERTK, PITPNC1	Reduces metastasis and angiogenesis	
	No specific target	Reduces tumorigenesis and metastasis	
**miR-140**	ALDH1/SOX9	Reduce stemm cell formation	37–39
	SOX2/SOX9	Inhibit stemm cell signaling	
	COL4A1, ITGA6, MARCKSL	Reduce cell proliferation and migration	
**miR-146b**	NFkB, IL-6/STAT3	Inhibit migration and invasion and metastasis	40–43
	FOXP3	Triggering apoptosis	
	BRMS1	Suppress breast cancer metastasis	
**miR-200c**	TGF-b, ZEB1/2, SNAIL1/2	Suppress EMT	44–50
	ELP1/HDAC2	Suppress EMT	
	KRAS	Inhibit tumor growth	
	PRKAR1A, PRKACB	Reduce migration	
**miR-206**	Cx43	Reduces migration, invasion and metastasis	51–56
	MKL1/IL11 pathway	Inhibits cancer cell stemness and metastasis	
	VEGF, MAPK3, and SOX9	Inhibit cell invasion and angiogenesis	
	Tbx3	Inhibit cell proliferation, invasion, and maintenance of the cancer stem cell population	
	TGF-β, NRP1, SMAD2	Suppresses EMT	
	CORO1C	Inhibit cell migration	
	ER-a	Suppress cell proliferation	
**miR-335**	SOX4, TNC	Suppresses metastasis and migration	33, 57–59
	SOX4, TNC	Selective metastasis suppressor and tumor initiation	
	BRCA1	Inhibit cell proliferation and activate apoptosis	

Briefly, miR-30a and miR-30c have been reported to inhibit cell migration and invasion through targeting vimentin or other epithelial mesenchymal transition (EMT)-related molecules such as Slug or TWIF1^20,23,27^. MiR-30a also regulates UBE3C, a ubiquitin protein ligase family, resulting in suppression of cell proliferation and migration^25^. MiR-31 demonstrates cell proliferative and invasive properties through miR-31-mediated down-regulation of WAVE3, GNA13, PRKCE, or RhoA^29–31,33,61^. MiR-126 and miR-335 are tumor suppressive miRNAs that reduce bone- or lung-metastasis using cell-based comprehensive miRNA microarray analysis in human breast cancer^34^. MiR-335 also suppresses metastasis and migration through targeting of the progenitor cell transcription factor SOX4 and extracellular matrix component tenascin C (TNC)^34^. MiR-140 promotes cancer stem cell formation in basal-like early stage breast cancer through miR-140/ALDH1/SOX9 axis^38^. Another group also reported that miR-140/SOX2/SOX9 axis regulates cancer stem cells in early breast cancer^39^. miR-140 also contributes to tumor suppressive effect by targeting COL4A1, ITGA6 and MARCKSL1 in breast cancer^40^. MiR-146b shows tumor suppressive function through the regulation of NF-κB-IL-6/STAT3 signaling pathway in breast cancer^41,43^. FOXP3-miR-146 family-NF-κB axis provides tumor suppressor function such as inhibition of cell growth or tumor metastasis *in vitro* or *in vivo* assay^44^. MiR-200c has been reported to show significant tumor suppressive function in several solid tumors including breast cancer^62^. Specifically, miR-200c suppresses TGF-β signaling pathway and targets ZEB1/2 or SNAIL1/2, resulting in inhibition of EMT in breast cancer^45,46,50,51^. MiR-200c was also reported to regulate EMT through PELP1/HDAC2^47^. MiR-200c inhibits breast cancer proliferation by targeting KRAS^49^. Lastly, miR-206/TWF1/MKL1-SRF/IL-11 signaling pathway inhibits breast cancer initiation and progression^56^.

### Expression levels of the 9 tumor-suppressive miRNAs in breast cancer tissue and paired normal breast tissue

**Figure 1:**
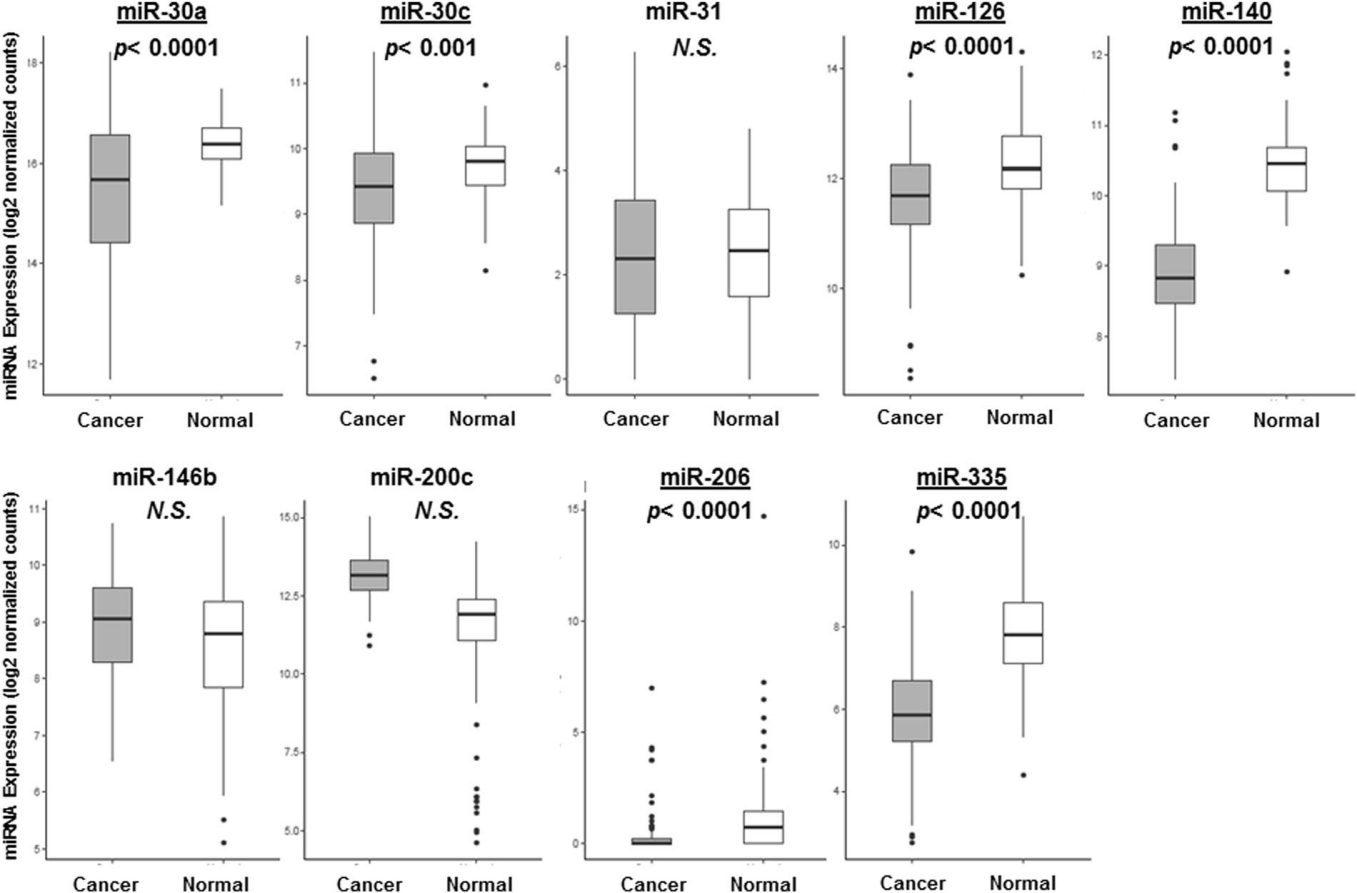
Expression levels of the 9 tumor-suppressive miRNAs in breast cancer samples and their paired normal breast samples retrieved from TCGA dataset (n = 103). One-sided p < 0.05 was considered statistically significant for analysis of expression levels in cancer *vs*. normal tissue (tested normal greater than tumor).

The expression levels of each of the 9 tumor suppressive miRNAs in breast cancer tissue were compared with their paired normal breast tissue using TCGA dataset (n = 103 each group). Of the 9 tumor suppressive miRNAs, only miR-30a, miR-30c, miR-126, miR-140, miR-206, and miR-335 were found to show significantly lower expression levels in breast cancer tissue compared with paired normal breast tissue (p < 0.0001, p < 0.001, p < 0.0001, p < 0.0001, p < 0.0001, and p < 0.0001, respectively). Unexpectedly, miR-31, miR-146b, and miR-200c did not show any significant differences (Fig. 1). Interestingly, miR-200c showed higher expression levels in cancer tissue than in normal tissue, which was an opposite trend from previous reports (Fig. 1).

### Prognostic relevance of the tumor suppressive miRNA expression in breast cancer patients

**Figure 2:**
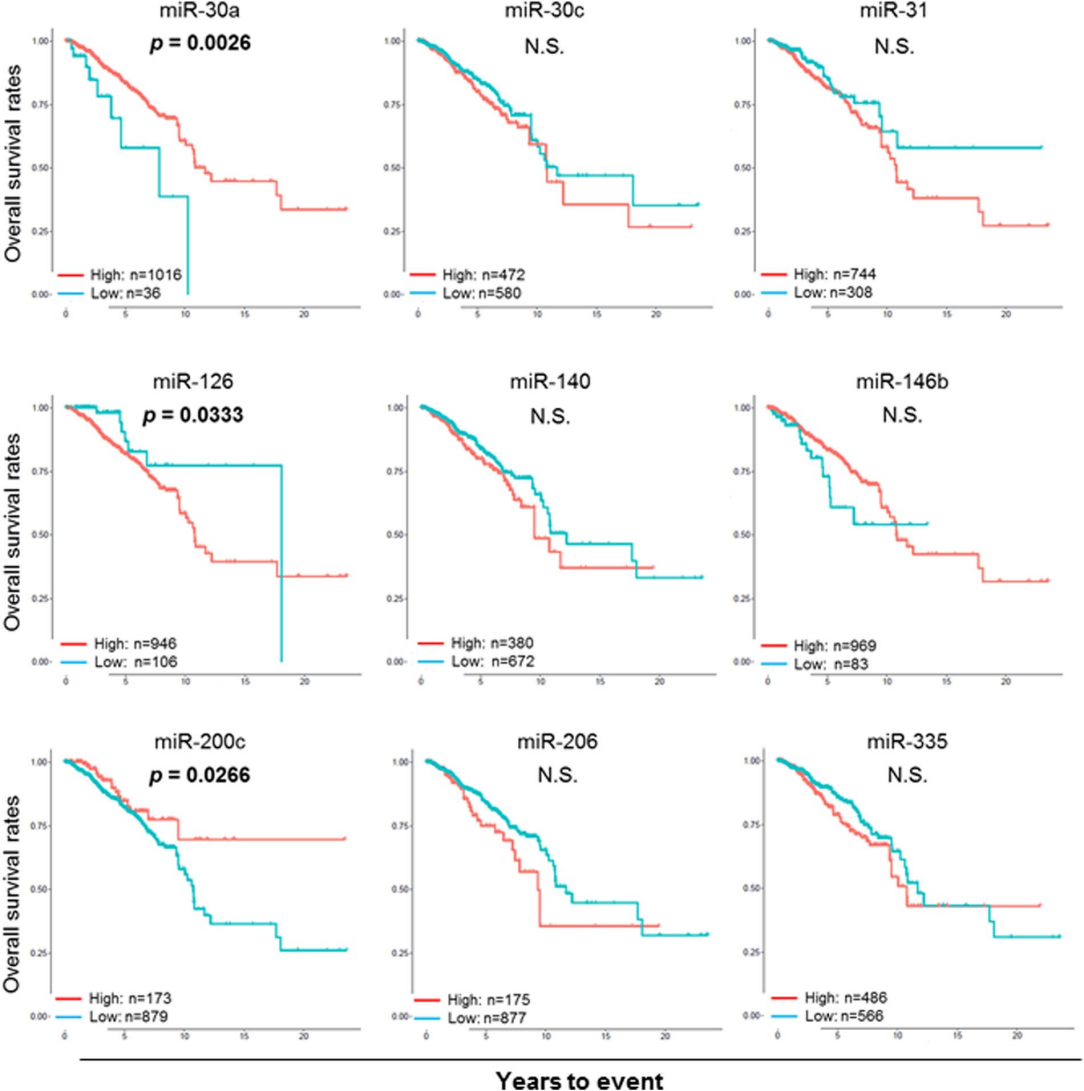
Expression of 9 selected tumor suppressive miRNAs in breast cancer was studied for their impact on overall survival (OS). OS was compared using the Kaplan-Meier curves and log rank test between the high (red line) and low (blue line) expression groups determined by each miRNA-specific thresholds. P value in bold type indicates statistical significance.

**Figure 3:**
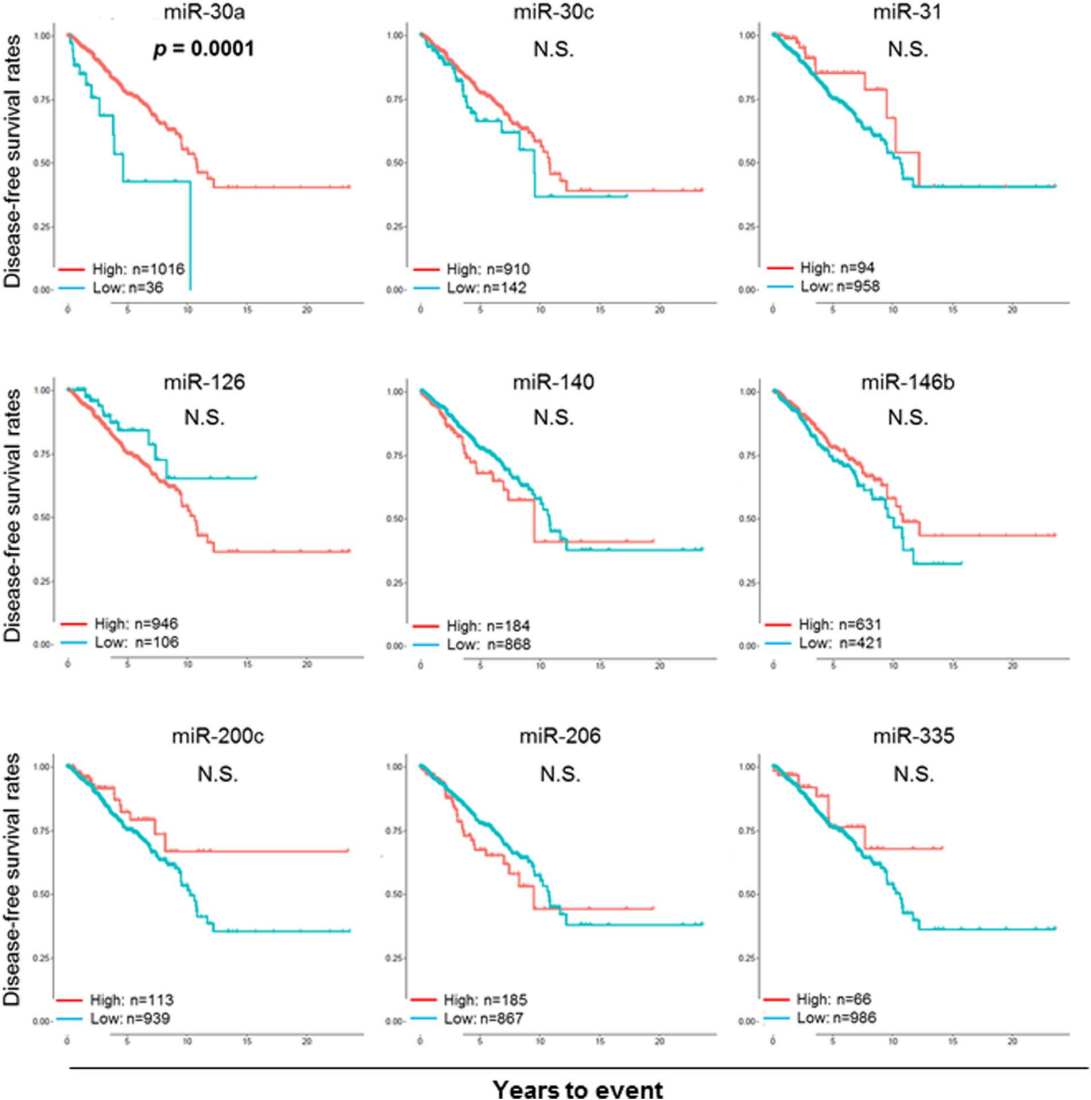
Expression of 9 tumor suppressive miRNAs in breast cancer was studied for their impact on patient’s disease-free survival (DFS). DFS was compared using the Kaplan-Meier curves and log rank test between the high (red line) and low (blue line) expression groups determined by each miRNA-specific thresholds. P value in bold type indicates statistical significance.

In order to determine the prognostic relevance of the 9 tumor suppressive miRNAs in breast cancer, OS was analyzed using the Kaplan-Meier curves and log rank test between the high and low expression groups of each miRNAs. The OS analysis demonstrated that high expression levels of miR-30a and miR-200c demonstrated significantly better survival (Log rank test, *p* = 0.0026 and *p* = 0.0266, respectively), while the other 6 miRNAs, miR-30c, miR-31, miR-140, miR-146b, miR-206, and miR-335, demonstrated no significant survival difference between high and low expression groups (Fig. 2). Surprisingly, high expression of miR-126 showed significantly worse prognosis (Log rank test, *p* = 0.0333) (Fig. 2), which was contrary to previous reports. As for the DFS analysis, high expression levels showed significantly better survival only for miR-30a (Log rank test, p = 0.0001), while high expression of miR-200c showed only a tendency toward better prognosis (Log rank test, p = 0.097) (Fig. 3).

### Survival analyses of the suppressive miRNAs in different stages and subtypes

**Figure 4:**
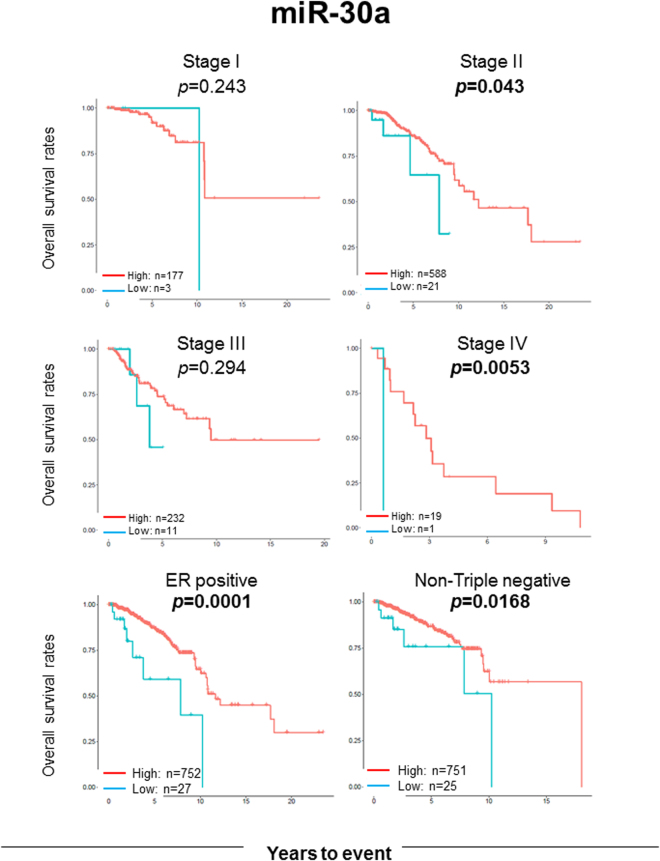
OS analyses of miR-30a in each stage and subtypes (ER positive and non-triple negative subgroups). OS was compared using the Kaplan-Meier curves and log rank test between the high (red line) and low (blue line) expression groups determined by the miRNA-30a-specific thresholds. P value in bold type indicates statistical significance.

**Figure 5:**
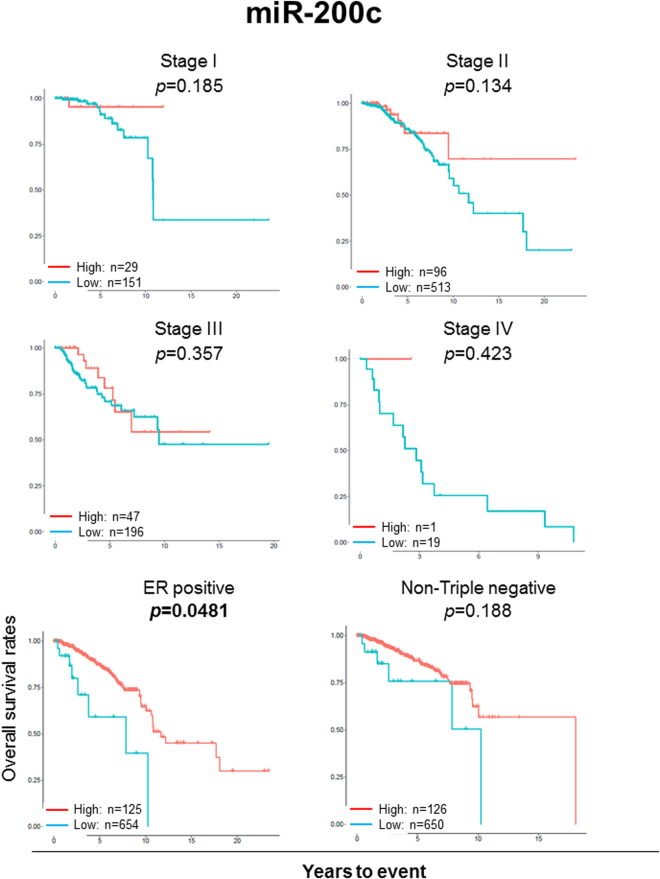
OS analyses of miR-200c in each stage and subtypes (ER positive and non-triple negative subgroups). OS was compared using the Kaplan-Meier curves and log rank test between the high and low expression groups determined by the miRNA-200c-specific thresholds. P value in bold type indicates statistical significance.

To further clarify which clinical stage and histology miR-30a and mir-200c demonstrate their tumor suppressive function, we conducted survival analyses on different TNM stages and subtypes for these 2 miRNAs. We found that high expression level of miR-30a was significantly associated with better survival in patients with stage II and IV cancer (p = 0.043 and 0.0053, respectively), and ER positive and non-triple negative subtypes (Log rank test, *p* = 0.0172, *p* = 0.0001, and *p* = 0.0168, respectively) (Fig. 4). Breast cancer is known to have two important subtypes, which have distinct signaling networks and drug targets^63^ and distinct prognostic signatures^64^. Therefore, we conducted survival analysis of luminal and basal-like subtypes of breast cancer based upon PAM50 classification. High expression level of miR-30a was significantly associated with better survival in both luminal and basal-like breast cancer subtypes (p = 0.0012 and p = 0.011, respectively) (Supplementary Figure S1). High expression of miR-200c was found to be significantly associated with better prognosis in patients with ER positive breast cancer. Patients with high expression of miR-200c demonstrated the trend towards better prognosis in both early and advanced stage breast cancer, but it was not statistically significant (Fig. 5).

Univariate and multivariate Cox regression stratified analyses in TCGA dataset were performed on 2 selected miRNAs: miR-30a and miR-200c. When clinical stage or hormonal subtypes were defined as covariates in Cox regression analyses, the 2 miRNAs demonstrated no significant differences (Supplementary Table S1).

### Association between EMT and TGF-β signaling related gene sets and miR-30a or miR-200c expression levels using GSEA

**Figure 6:**
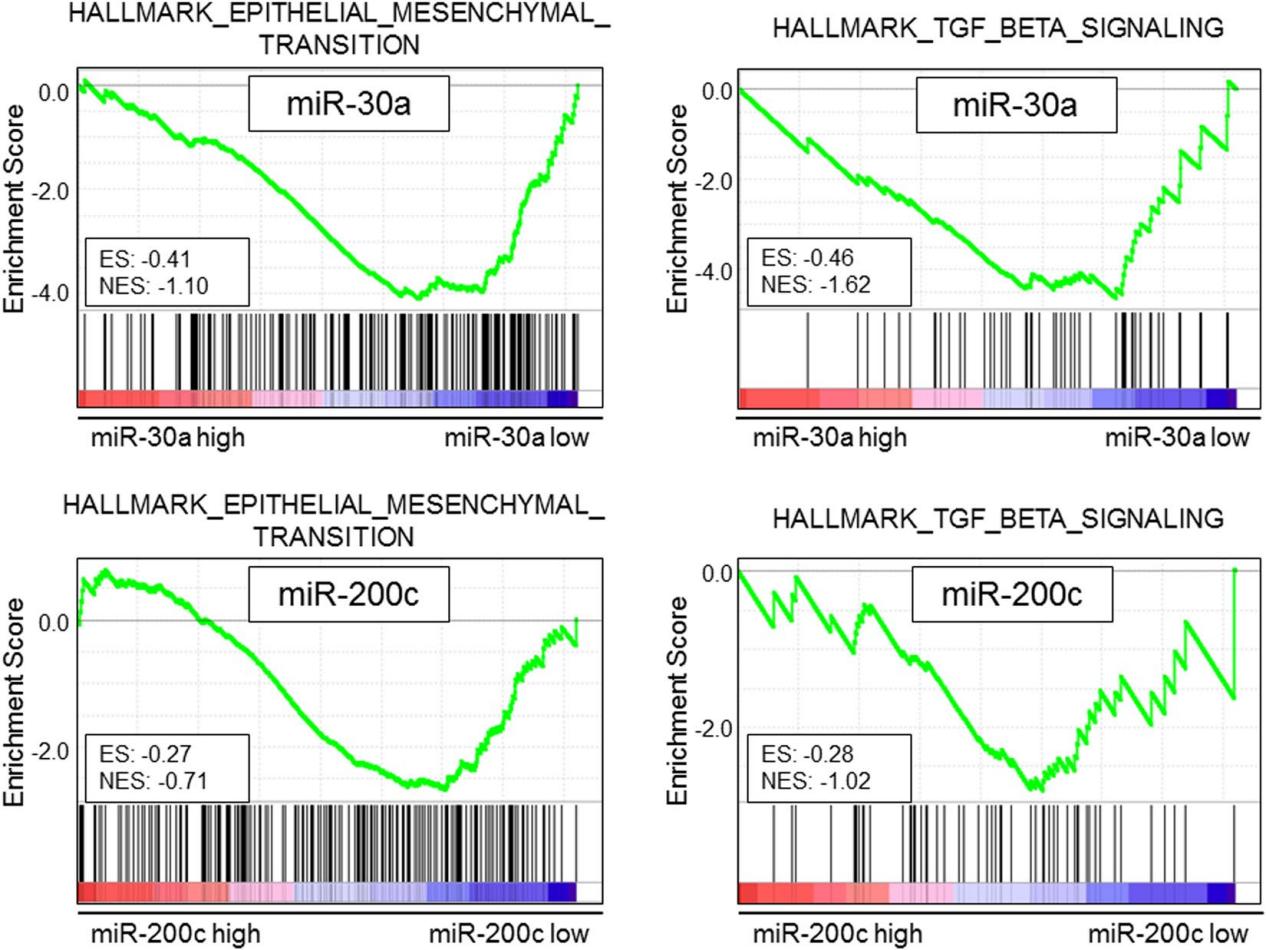
GSEA for expression levels of miR-30a or miR-200c. GSEA analyses were performed for HALLMARK EPITHELIAL MESENCHYMAL TRANSITION and HALLMARK TGF BETA SIGNALING using TCGA. ES, enrichment score; NES, normalized enrichment score.

MiR-30a and miR-200c, which demonstrated significant survival associations in TCGA cohort, were previously demonstrated to have tumor suppressive function through EMT in breast cancer (Supplementary Figure S2). Therefore, we conducted GSEA to validate whether the miRNA expression levels were associated with EMT using TCGA cohort, as well as EMT-related gene set of transforming growth factor-β (TGF-β) signaling. Predefined gene sets of the HALLMARK_EPITHELIAL_MESENCHYMAL_TRANSITION and HALLMARK_TGF_BETA_SIGNALING from GSEA, previously described to be involved in EMT and TGF-β, were used in this analysis. Interestingly, low expression levels of miR-30a showed a tendency with high enrichment score of EMT gene set (ES: -0.41, NES: -1.10, p = 0.393) and TGF-β signaling gene set (ES: -0.46, NES: -1.62, p = 0.053), while low expression levels of miR-200c did not show any association in TCGA cohort (EMT, ES: -0.27, NES: -0.71, p = 0.754; TGF-β signaling, ES: -0.28, NES: -1.02, p = 0.450) (Fig. 6).

It is important to examine whether miR-30a or miR-200c targets are associated with Cancer Hallmark-associated gene sets; apoptosis, cell cycle, cell death, cell motility, DNA repair, immune response, and phosphorylation^65,66^. The GSEA of C5 demonstrated that some gene sets are significantly associated with miR-30a and miR-200c expression. Several gene sets related to cell cycle, DNA repair, or phosphorylation are associated with miR-200c expression. Some gene sets related to phosphorylation are associated with miR-30a expression (Supplementary Dataset S1). Although the evidence is not robust enough, the result of the GSEA suggests that miR-30a and miR-200c might play potential roles in cancer biology linked to Cancer Hallmark-associated gene sets.

## Discussion

During the last decade, many studies have demonstrated the individual biodiversity of breast cancer such as the intrinsic subtypes based on genome wide analyses^67–69^, and other sources of breast cancer heterogeneity^70–72^. Clinical classifications have been used to estimate the efficacy of hormonal therapy or molecular targeted therapy in clinical settings based on these analyses^68,69^. For instance, the luminal subtypes were generated through coding-gene expression. On the other hand, ncRNA expression has not been as extensively studied. In 2005, Iorio *et al*. first demonstrated that several miRNAs were deregulated in human breast cancer using miRNA microarray^12^ and many reports have been published in that field since then. Although many tumor-related miRNAs, including oncogenic miRNAs and tumor suppressive miRNAs, have been elucidated as prognostic biomarkers in breast cancer, few of them have been validated using large cohorts.

TCGA dataset includes comprehensive genetic and epigenetic information, as well as clinical data such as age, gender, race, pathological diagnosis, survival, and tumor recurrence of over 1000 breast cancer patients. To date, few reports have utilized this public dataset as a validation cohort for tumor suppressive miRNA in breast cancer. Therefore, we first conducted a systematic literature search and determined promising candidates of tumor suppressive miRNA as prognostic biomarkers in breast cancer. We then evaluated their clinical relevance using TCGA cohort. We analyzed 9 tumor suppressive miRNAs (miR-30a, miR-30c, miR-31, miR-126, miR-140, miR-146b, miR-200c, miR-206, and miR-335) all previously reported in breast cancer. To our surprise, we found that only 2 out of the 9 selected miRNAs (miR-30a and miR-200c) demonstrated prognostic significance in TCGA cohort.

MiR-30a has been reported to target EMT-related molecules (such as vimentin or Slug) and to suppress tumor cell migration and invasion in breast cancer^20,23^ as well as other solid cancers^73^. MiR-30a has also been demonstrated to inhibit several critical oncogenes, such as Eya2, ITGB3, or UBE3C, and suppress tumor growth, cell migration and invasion^21,22,24,25^. MiR-200c is one of the most well-known tumor suppressive miRNAs in cancer. Hurteau *et al*. first demonstrated that overexpression of the miR-200c leads to reduced expression of ZEB1 and increased expression of E-cadherin in breast cancer cell lines^45^. Further, many reports have demonstrated that miR-200c has tumor suppressive functions related to EMT in breast cancer^46,47,50,51^ and other solid cancers^74–77^. However, the clinical relevance of miR-30a and miR-200c as prognostic biomarkers has never been investigated using large cohorts. In the present study, we found that high expression levels of miR-30a or miR-200c were associated with better OS and DFS in breast cancer using TCGA cohort. To our knowledge, this is the first report that demonstrates the prognostic relevance of tumor suppressive miRNAs in breast cancer patients using a sufficiently large cohort.

We recognize that there are limitations with our study. The TCGA dataset was collected from multiple institutes, which may introduce selection biases into our methods. There was also missing data such as some patients in TCGA cohort lacked clinical data. Further, although TCGA has a large sample size of patients with breast cancer, the number of patients with paired normal breast tissue was significantly smaller, which may have hindered the statistical power for that analysis.

In conclusion, we found that high expression of 2 tumor-suppressive miRNAs, miR-30a and miR-200c, was associated with better OS, whereas miR-30c, miR-31, miR-126, miR-140, miR-146b, miR-206, and miR-335 was not. To our knowledge, this is the first report that elucidated the feasibility of utilizing a publicly available database, such as TCGA, to validate the clinical relevance of tumor suppressive miRNA for patients with breast cancer.

## Materials and Methods

### Literature search to identify well established tumor suppressive miRNAs in breast cancer

We conducted a literature search using PubMed Central between 2005 and 2016 to identify well established tumor suppressive miRNAs in breast cancer. The criteria for selection were: 1) at least two research groups have demonstrated that the selected miRNA possesses only tumor suppressive function (and not oncogenic function) both *in vitro* and *in vivo*, 2) the target mRNA or signaling pathway of the miRNA have been identified in breast cancer, and 3) the clinical relevance of miRNA has yet to be elucidated using a large cohort of breast cancer patients.

### Extraction of miRNA-Seq and clinical dataset from TCGA

All data including the expression levels of the miRNAs of interest (miRNA-Seq) and clinical data were obtained from TCGA breast cancer cohort through the Genomic data common (GDC) data portal. The survival data of the breast cancer patients in the TCGA was obtained as previously reported^19^. Among the 1,097 patient breast cancer samples logged in TCGA, 1,052 samples that had both miRNA-Seq data and survival information were used in this study. Since TCGA is a collection of de-identified publically accessible database, Institutional Review Board review was waived.

### Comparison of miRNAs expression levels between breast cancer and paired normal breast tissue using TCGA cohort

To evaluate the expression level of each candidate tumor suppressive miRNA in breast cancer tissue, the miRNA-Seq expression quantification data of breast cancer tissue (n = 103) and the paired normal breast tissue (n = 103) were retrieved from the GDC data portal.

### Prognostic analysis of the tumor suppressive miRNAs using TCGA cohort

Overall survival (OS) was defined as the time from the date of diagnosis to the date of death by any cause, and disease-free survival (DFS) was defined as the time from the date of diagnosis to the date of diagnosis of a recurrent breast cancer. Patients who did not have an event were censored at the last date of follow-up or after 10 years from clinical records. OS or DFS was compared using the Cox proportional hazard model between expression groups (high versus low) determined by each miRNA-specific thresholds. Namely, differences in the OS between the two groups were assessed at multiple candidate cutoff points within the range of observed expression value, and the optimal cut point was chosen based on the statistical significance of the Cox proportional hazard model. Stratified analyses were also performed. The covariates in the models included tumor TNM stage (American Joint Commission on Cancer Clinical Cancer Staging 7^th^ edition), estrogen receptor (ER), progesterone receptor (PR), and HER2 status. In TCGA data set, the histological subtypes were determined using pathological molecular subtyping^78,79^. Univariate and multivariate Cox regression stratified analyses for OS were also conducted and the covariates in the models included tumor TNM stage, ER, PR, HER2 status, and the expression levels of each miRNA of interest.

### Gene Set Enrichment Analysis (GSEA) for miRNA expression

To investigate whether the miRNAs of interest had significant associations with metastasis-related gene sets, GSEA was conducted with the miRNAs of interest and mRNA expression data from TCGA. GSEA was performed using software provided by the Broad Institute (http://software.broadinstitute.org/gsea/index.jsp)^80^. We performed GSEA for Hallmark gene sets, which summarized and represented specific well-defined biological states or processes and displayed coherent expression.

### Statistical analysis

All statistical analyses were performed using R software (http:///www.r-project.org/) and Bioconductor (http://bioconductor.org/). Data of miRNA expression was normalized using DESeq. 2 package^81^ and log-transformed. Patients were dichotomized into low-expression group and high-expression groups based on the miRNA expression levels. A running Cox proportional hazard statistics was applied to determine the threshold of the dichotomization^82^. To compare the survival curves of individual groups, the Kaplan-Meier method with log-rank tests and Cox proportional hazard models were used when appropriate. To test the proportional Hazard assumption in Cox models, Schoenfeld residuals test was used. The reported results included hazard ratios (HR) and 95% confidence intervals (CI). One-sided p < 0.05 was considered statistically significant for analysis of expression levels in cancer *vs*. normal tissue (tested normal greater than tumor), and two-sided p < 0.05 was considered statistically significant for survival analysis.

## Electronic supplementary material

Supplementary information

Supplementary Dataset
