# Supplementary material for: Overexpression of suppressive microRNAs, miR-30a and miR-200c are associated with improved survival of breast cancer patients
Source: Sci Rep. 2017 Nov 21;7:15945. doi: 10.1038/s41598-017-16112-y (PMC5698306; doi:10.1038/s41598-017-16112-y)
Supplement: Supplementary file 1 — Supplementary information [file 41598_2017_16112_MOESM1_ESM.pdf]

Supplementary information

Title:

Overexpression of suppressive microRNA, miR-30a and miR-200c is associated with improved the survival of breast cancer patients

Tsutomu Kawaguchi, Li Yan, Qianya Qi, Xuan Peng, Emmanuel M. Gabriel, Jessica Young, Song Liu, Kazuaki Takabe

Supplementary Table S1

OS

miR-200c

**Variables**	**Univariate model**				**Multivariate model**			
	**HR**	**Lower**	**Upper**	**P-value**	**HR**	**Lower**	**Upper**	**P-value**
**hsa-mir-200c**	0.92	0.82	1.03	0.14	0.91	0.81	1.02	0.1
**Stage**	2.24	1.79	2.81	<0.0001	2.26	1.8	2.83	<0.0001
**hsa-mir-200c**	0.92	0.81	1.03	0.15	0.92	0.82	1.04	0.170
**ER**	0.63	0.44	0.93	0.018	0.63	0.43	0.92	0.016
**hsa-mir-200c**	0.91	0.81	1.03	0.13	0.92	0.82	1.04	0.206
**PR**	0.67	0.47	0.96	0.028	0.68	0.48	0.96	0.029
**hsa-mir-200c**	0.94	0.8	1.11	0.46	0.93	0.79	1.1	0.40
**HER2**	1.04	0.68	1.59	0.84	1.05	0.69	1.6	0.83

miR-30a

Variables	Univariate model				Multivariate model			
	HR	Lower	Upper	P-value	HR	Lower	Upper	P-value
**hsa-mir-30a**	0.96	0.84	1.1	0.58	0.98	0.85	1.12	0.73
**Stage**	2.24	1.79	2.81	<0.0001	2.24	1.79	2.81	<0.0001
**hsa-mir-30a**	0.99	0.87	1.14	0.94	1	0.86	1.15	0.947
**ER**	0.63	0.44	0.93	0.018	0.63	0.44	0.93	0.018
**hsa-mir-30a**	1	0.87	1.15	0.99	0.99	0.86	1.14	0.868
**PR**	0.67	0.47	0.96	0.028	0.67	0.47	0.96	0.028
**hsa-mir-30a**	0.95	0.81	1.11	0.52	0.95	0.81	1.1	0.48
**HER2**	1.04	0.68	1.59	0.84	1.05	0.69	1.6	0.83

DFS

miR-200c

Variables	Univariate model				Multivariate model			
	HR	Lower	Upper	P-value	HR	Lower	Upper	P-value
**hsa-mir-200c**	0.95	0.84	1.07	0.38	0.94	0.84	1.05	0.28
**Stage**	2.19	1.78	2.69	<0.0001	2.21	1.79	2.72	<0.0001
**hsa-mir-200c**	0.96	0.85	1.08	0.5	0.96	0.86	1.09	0.56
**ER**	0.69	0.49	0.97	0.031	0.69	0.49	0.96	0.03
**hsa-mir-200c**	0.95	0.85	1.07	0.44	0.97	0.86	1.09	0.623
**PR**	0.69	0.5	0.95	0.021	0.69	0.51	0.95	0.021
**hsa-mir-200c**	1.02	0.87	1.21	0.77	1.01	0.86	1.19	0.86
**HER2**	1.19	0.82	1.71	0.36	1.19	0.82	1.71	0.36

miR-30a

Variable	Univariate model				Multivariate model			
	HR	Lower	Upper	P-value	HR	Lower	Upper	P-value
**hsa-mir-30a**	0.93	0.83	1.05	0.24	0.95	0.84	1.07	0.36
**Stage**	2.19	1.78	2.69	<0.0001	2.19	1.78	2.69	<0.0001
**hsa-mir-30a**	0.96	0.85	1.08	0.48	0.96	0.85	1.08	0.476
**ER**	0.69	0.49	0.97	0.031	0.69	0.49	0.97	0.031
**hsa-mir-30a**	0.97	0.86	1.09	0.57	0.95	0.84	1.07	0.43
**PR**	0.69	0.5	0.95	0.021	0.69	0.5	0.94	0.02
**hsa-mir-30a**	0.92	0.8	1.05	0.2	0.91	0.8	1.04	0.15
**HER2**	1.19	0.82	1.71	0.36	1.2	0.83	1.72	0.33

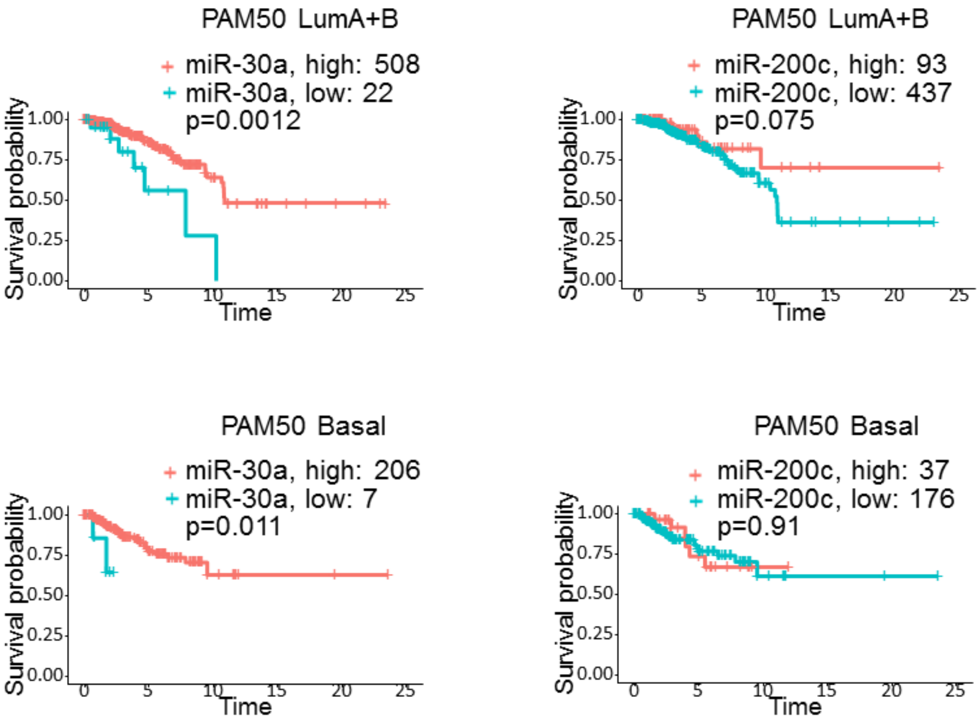

Supplementary Figure S1

OS analyses of miR-30a and miR-200c in each subtypes using PAM50 classification. OS was compared using the Kaplan-Meier curves and log rank test between the high and low expression groups determined by the each miRNA-specific thresholds.

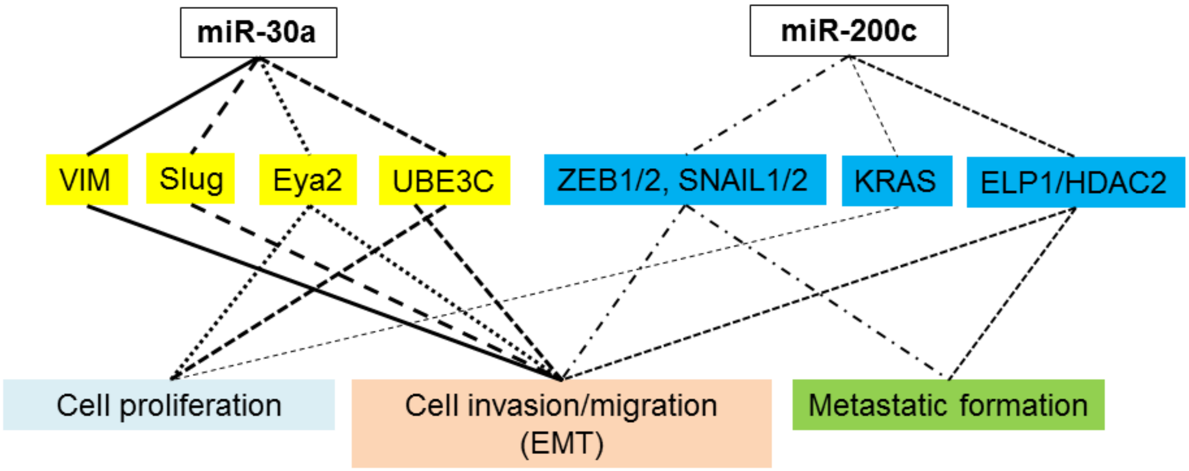

Supplementary Figure S2

MiR-30a has been reported to target EMT-related molecular (such as Vimentin or Slug) and to suppress tumor cell migration and invasion in breast cancer, as well as other solid cancers. MiR-200c has been reported to show tumor suppressive function through EMT-related molecular such as ZEB1/2 or SNAIL1/2.
